# Structural characteristics mediate forest mitigation potential against climate change and biodiversity loss

**DOI:** 10.1002/eap.70211

**Published:** 2026-03-16

**Authors:** Julian Lunow, Sabina Burrascano, Lorenzo Balducci, Francesco Chianucci, Lucas Chojnacki, Inken Doerfler, Jeňýk Hofmeister, Jan Hošek, Péter Ódor, Peter Schall, Tommaso Sitzia, Nadja K. Simons

**Affiliations:** ^1^ Biocenter, Chair of Conservation Biology and Forest Ecology, Applied Biodiversity Science University of Würzburg Würzburg Germany; ^2^ Institute of Ecology Leuphana University Lüneburg Lüneburg Germany; ^3^ Department of Environmental Biology Sapienza University of Rome Rome Italy; ^4^ CREA Research Centre for Forestry and Wood Arezzo Italy; ^5^ Forest & Nature Lab Ghent University Ghent Belgium; ^6^ Department of Plants and Crops, UAV Research Centre Ghent University Ghent Belgium; ^7^ Department of Plant Sociology and Nature Conservation Carl von Ossietzky University of Oldenburg Oldenburg Germany; ^8^ Faculty of Forestry and Wood Sciences, Department of Forest Ecology Czech University of Life Sciences Prague Prague Czech Republic; ^9^ Ecological Services Hořovice Czech Republic; ^10^ Centre for Ecological Research Institute of Ecology and Botany Vácrátót Hungary; ^11^ Institute of Environmental Protection and Nature Conservation University of Sopron Sopron Hungary; ^12^ Silviculture and Forest Ecology of the Temperate Zones University of Göttingen Göttingen Germany; ^13^ Department Land, Environment, Agriculture and Forestry Università degli Studi di Padova Legnaro Italy

**Keywords:** biodiversity conservation, carbon storage, climate change, deadwood quality, forest management, multi‐taxon

## Abstract

European forests play an important role for climate change mitigation and biodiversity conservation. As they have been shaped by silviculture for centuries, it is important to understand how management practices affect forest structure and in turn influence the role of forests in achieving both goals. We analyzed data on a wide range of temperate European forests encompassing the most widespread management regimes to understand the interplay of forest structure, aboveground carbon stocks, and the richness of several taxonomic groups. Using structural equation modeling, we identified the forest structural characteristics that are positively correlated with both carbon stocks and species richness. We found that stand age and tree species richness are related to other forest structural characteristics, which had positive links to carbon stocks in deadwood. Increasing stand age was associated with an increase in deadwood carbon stocks. There were no direct negative relationships between stand age or tree species richness and the richness of different taxonomic groups. An increasing richness of deadwood types had positive links with the species richness of birds, saproxylic beetles, and saproxylic fungi, as with deadwood carbon stocks. However, increases in the species richness of birds and understory vascular plants were negatively related to increasing carbon stocks in living wood, while beetle species richness was positively related to this carbon stock. Birds' species richness was directly and positively associated with increasing mean tree diameter. Conversely, a higher richness of tree species was indirectly linked to lower carbon stocks in living wood. Additionally, an increase in mean tree diameter was indirectly correlated with a decrease in bird and vascular plant species richness. Our findings highlight potential trade‐offs between carbon stocks in living wood and the species richness of several taxonomic groups in European forests, while the species richness of some taxonomic groups was positively correlated to deadwood carbon stocks. Policies focused on increasing living biomass may not target both the climate and biodiversity crises. Instead, the diversity of deadwood emerges as a key factor in explaining the relationship between carbon storage and biodiversity, and should hence play a prominent role in forest management strategies and related policies.

## INTRODUCTION

Forest ecosystems play a key role in climate change mitigation as they globally store large amounts of carbon (861 ± 66 Pg carbon) in the soil (44%), in above‐ and belowground live biomass (42%), in deadwood (8%), and in litter (5%) (Canadell & Raupach, 2008; Pan et al., 2011). In addition, forests are gaining in relevance for climate change mitigation through their role in substitution of fossil fuels with woody biomass (Sasaki, 2021). Biodiversity plays a key role in both processes by driving the quantity and quality of ecosystem processes (e.g., carbon storage, timber production) through its influence on ecosystem functioning (Chapin III et al., 2000; Hisano et al., 2018; Pardos et al., 2021) and resilience (Lipoma et al., 2024). As biodiversity is continuously threatened by the impacts of human activities on habitats (e.g., land use) and by environmental changes (Pereira et al., 2010; Sala et al., 2000), the uncertainties for ecosystem functioning and resilience keep increasing. Given the close link between the climate and biodiversity crises, it is crucial to define policies and actions that address both simultaneously (Burrascano et al., 2016).

At a global level, synergies between tree species diversity and forest productivity and thus higher carbon storage potential were found (Jactel et al., 2018; Zhang et al., 2012). Not only does the rate of tree carbon accumulation increase with tree size (Lutz et al., 2018; Stephenson et al., 2014), we also see an additional positive relationship between the diversity of trees and carbon stocks (Cavanaugh et al., 2014; Sullivan et al., 2017). This is based on the positive effect of forest stand age on both aboveground biomass and tree diversity (Lasky et al., 2014). In tropical regions, old growth forests have a higher diversity of amphibians, birds, and mammals and high carbon stocks due to higher structural complexity and better carbon storage potential compared to younger forest stands (Armenteras et al., 2015; Deere et al., 2018; Strassburg et al., 2010). Comparing forests with different management focus at a global level, Di Marco et al. (2018) found that management that enhances carbon sequestration also increased biodiversity of amphibians, birds, and mammals. However, trade‐offs are found at large spatial extents as several biodiversity hotspots are located in regions with low carbon stocks (Phelps et al., 2012; Strassburg et al., 2010), and also at smaller spatial extents (Di Marco et al., 2018). Trade‐offs between biodiversity conservation and aboveground carbon storage have also been found in temperate European forests on the level of single forest stands, with no or weak links between the carbon stocked in tree biomass and biodiversity (Asbeck et al., 2021; Sabatini et al., 2019). Knowledge about the relationship between carbon storage and biodiversity on large spatial extents outside tropical regions is lacking, especially for some taxonomic groups, such as insects, vascular plants, and fungi. To inform environmental policies, it is important that we increase our knowledge on correlations between carbon and biodiversity at different geographical scales.

In relation to the relationship of aboveground carbon storage and the structural characteristics and complexity of forests, Yuan et al. (2018) found a positive association through niche differentiation and facilitation. Furthermore, large trees play a significant role in forest carbon storage due to their substantial wood volume (Lutz et al., 2018; Mildrexler et al., 2020). The high volume of living wood in unmanaged and undisturbed forests also leads to an increase in deadwood through the natural aging and succession of trees (Oettel et al., 2020). However, the carbon storage potential of deadwood is rarely considered in forest management strategies aimed at enhancing carbon storage. Both living and dead wood biomass provide essential habitats for different species (Benedetti et al., 2021; Paillet et al., 2017; Seibold et al., 2015). The impact of living wood on biodiversity is influenced by the density of tree‐related microhabitats (TreMs), canopy cover, tree diameter at breast height (dbh), and the number of vertical strata (Basile et al., 2020; Benedetti et al., 2021; Larrieu et al., 2019; Tinya et al., 2021). A higher volume but also diversity of deadwood positively affects the species richness of various taxonomic groups, particularly saproxylic ones (Lassauce et al., 2011; Sandström et al., 2019; Seibold et al., 2015; Topp et al., 2006). Furthermore, species richness is related to the mass of aboveground living and dead wood through the various characteristics of forest structures formed by living and dead wood (Larrieu et al., 2017; Lutz et al., 2018; Tinya et al., 2021). This highlights the importance of the mass of both living and dead wood in supporting and maintaining forest biodiversity.

In this study, we are focusing on birds, saproxylic fungi, saproxylic beetles, and understory vascular plants (hereafter vascular plants) as indicators of forest biodiversity and their important role in ecosystem functioning (Xu et al., 2020; Zhou et al., 2023). Insect abundance, diversity, and biomass are influenced by different forest attributes such as canopy openness, deadwood volume and stand structural complexity (Rappa et al., 2022; Staab et al., 2023). Increasing deadwood volume has an especially positive effect on saproxylic beetles (Bouget et al., 2013; Doerfler et al., 2018; Sandström et al., 2019). But it is not only the amount of the carbon resources (i.e., deadwood volume) but also its diversity which has a pivotal role for insects (Brunbjerg et al., 2020; Lassauce et al., 2011). Fungal and vascular plant species richness is mainly positively correlated with stand age (Zeller et al., 2023). Furthermore, European forests dominated by trees native to Europe host the highest species richness of vascular plants, while this is only predominantly the case for fungi (Buée et al., 2011; Da Silva et al., 2019). For birds, important forest attributes driving their abundance are the dbh of living and dead trees and the amount of lying deadwood (Basile et al., 2021). In addition, the abundance and richness of birds is positively associated with increasing tree richness (May‐Uc et al., 2020). But relationships between forest structures and biodiversity, the response of different taxa and their community composition to changes in forest management often also differ between forest types (Da Silva et al., 2019; Leidinger et al., 2021; Storch et al., 2023). These complex relationships between forest structural characteristics, carbon stocks in different compartments, and the diversity of various taxonomic groups make it crucial that we understand the links between these parameters.

Here, we combined data on carbon stocks, forest structural attributes, and multi‐taxon richness from a range of forest types across Europe to address the following questions:

1. How do forest structural attributes influence aboveground carbon stocks and the species richness of different taxonomic groups? We expect that forest attributes directly and indirectly affect carbon stocks and the richness of different taxonomic groups.
2. Do carbon stocks in living and dead wood impact the species richness of different taxonomic groups? We expect that carbon stocks mediate the indirect effects of forest attributes on the different taxonomic groups and have direct effects on them.

## METHODS

### Study sites

**FIGURE 1:**
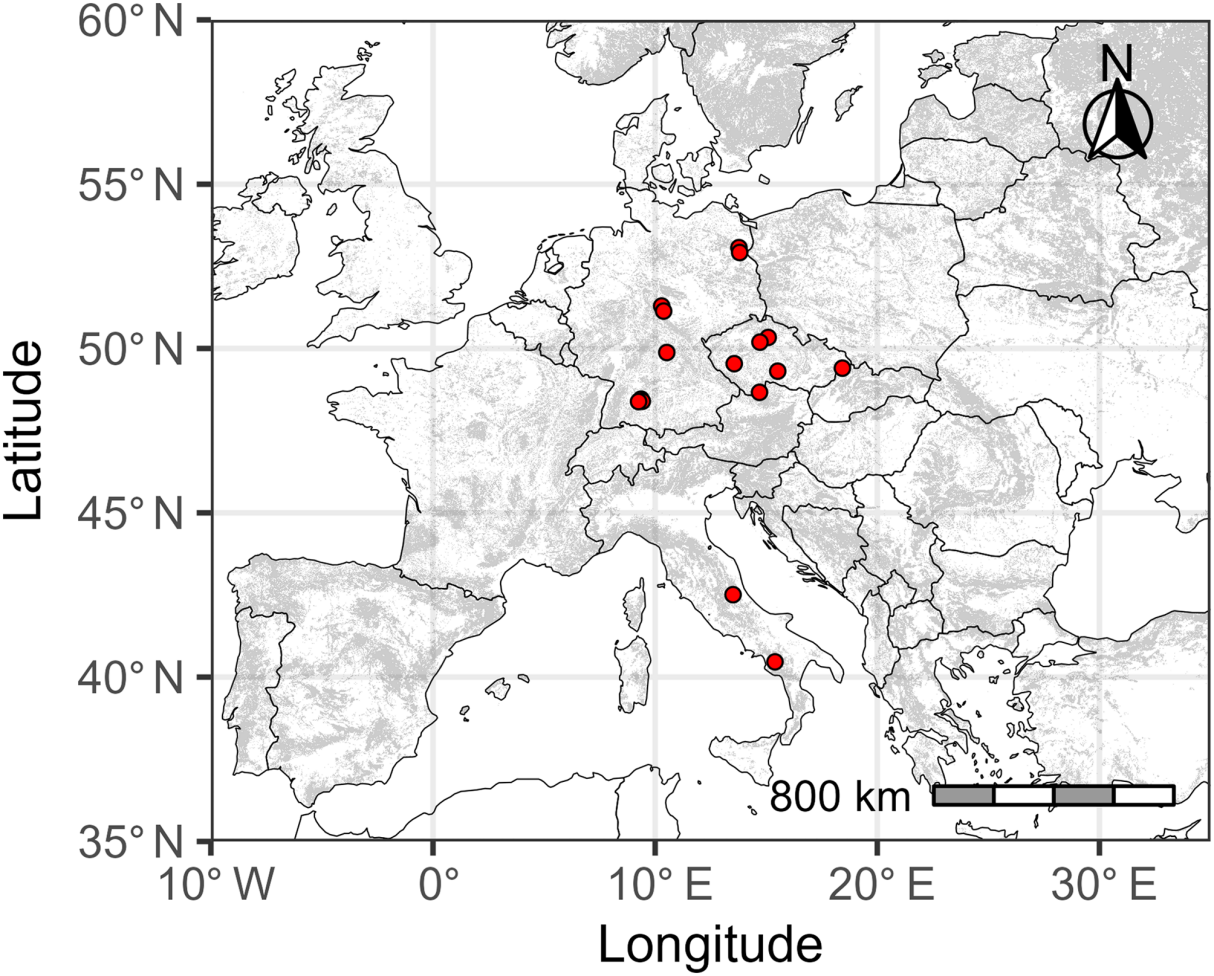
Locations of the considered sites (red dots) from the BOTTOMS‐UP database (https://www.bottoms‐up.eu/en/results/data‐explorer.html) in Central Europe. Each site includes 3–49 forest stands. Gray areas are covered by forests with a tree cover greater than 40% according to the map of Kempeneers et al. (2011).

We combined data on carbon stocks, forest structural characteristics, and multi‐taxonomic biodiversity from the BOTTOMS‐UP database (https://www.bottoms-up.eu/en/results/data-explorer.html), which gathered a comprehensive knowledge of European multi‐taxonomic forest biodiversity through the collaboration of research groups that collected data locally (Burrascano et al., 2023). Out of 3625 plots from 12 countries in the database, we selected 301 plots, covering a wide geographical range of temperate Europe. The plots are distributed from Italy (23 plots) over the Czech Republic (79 plots) to Germany (199 plots) (Figure 1). Across these countries, the plots are grouped into 16 sites that encompass different climatic conditions (Appendix S1: Table S1). The dataset covers different forest types (hemiboreal forest and nemoral coniferous and mixed broadleaved‐coniferous forest, mesophytic deciduous forest, beech forest, mountainous beech forest, plantations, and self‐sown exotic forests) and several widespread silvicultural strategies (shelterwood, retention clearcutting, simple clearcutting, and selection cutting) (Trentanovi et al., 2023) (Appendix S1: Table S2).

### Forest structure and carbon stock assessment

On each plot, a tree inventory was performed for all standing living trees. For each individual tree, the species was identified, and the diameter was measured. Since the data were collected through different protocols, we harmonized the data on the living trees by including only trees with dbh equal to or larger than 11 cm, and by extrapolating to 1 ha. We derived the tree species richness and the mean tree dbh at the plot level. In addition, we defined the age of the stand as the number of years after planting or after the last regeneration cut. Tree height was either measured or derived from the available measures for each specific tree (including unit height, unit diameter, and unit ID), while also incorporating information about the plot, site, and forest category by using allometric tree‐height models and the predictive mean matching method of the “mice” function from the “mice” package (Van Buuren & Groothuis‐Oudshoorn, 2011). To calculate the carbon stocks of living trees per unit (t/ha), we determined the single tree volume from tree heights and diameters. This volume was then multiplied by the wood basic density for each tree species to calculate the biomass. When the wood basic density was not provided with the original datasets, it was taken from the TRY database (Kattge et al., 2011). To calculate the carbon stocks of living trees per unit (t/ha), the biomass was multiplied by 0.48 (IPCC, 2003, 2006; Muukkonen, 2007). Tree dbh and height values for standing dead trees were handled in the same way as living trees. Across the study regions, lying deadwood pieces were measured within different thresholds in one or two diameters and length/height (Burrascano et al., 2021). These data were harmonized using a 15‐cm threshold. In addition, the decay stage (stage 1: undecayed, stage 2: weakly decayed, Stage 3: medium decayed, stage 4: very decayed, and stage 5: almost decomposed) and the deadwood type (log, stump) were determined and harmonized based on different decay stage protocols (Hunter, 1990; Kahl, 2008; Müller et al., 2007). For the calculation of deadwood type richness, we defined different diameter classes for the deadwood pieces (class 1: 15–25 cm, class 2: 25–35 cm, class 3: 35–50 cm, and class 4: >50 cm). The deadwood type richness used in our analysis is the number of unique combinations of diameter class, deadwood type (log, stump, and snag), and the decay stage (each unique combination can be considered equivalent to a “species”) per plot according to Siitonen (2001). To calculate the volume of the lying deadwood, we used the truncated cone formula. For the standing deadwood, the cone formula multiplied by 0.3 was used. Both were multiplied by the wood basic density with correction for decay class, deadwood type and if the deadwood piece belongs to a coniferous or broadleaved tree species (Appendix S1: Table S3; Di Cosmo et al., 2013). After that, we multiplied the output by 0.485 to compute the carbon stock of deadwood in tons per hectare (Martin et al., 2021).

Harmonizing the data from the different study plots in the database resulted in the creation of an artifact: the analyzed data only relates to even‐aged forests because in uneven‐aged forests, management interventions do not affect the whole stand and therefore the time since last intervention (which we used to define stand age) would not be directly comparable to even‐aged forests. The combination of tree richness, stand age, mean tree dbh, and deadwood type richness will hereafter be referred to as “forest structural characteristics.” All variables differed between plots (Table 1).

**TABLE 1:** Ranges (minimum, median, and maximum) and units of the forest structural characteristics, carbon stocks, and weather conditions included in the analysis.

Variable	Unit	Minimum	Median	Maximum
Stand age	year	5	111	212
Tree species richness	count/ha	1	2	14
Mean tree dbh	cm	12	33	102
Deadwood type richness	count/ha	1	11	25
C living wood	t/ha	0.545	103.398	361.536
C deadwood	t/ha	0.006	2.974	166.937
Mean annual temperature	°C	5.95	8.15	9.75
Annual precipitation	mm	557.1	821.3	1195.1

Abbreviations: C, carbon; dbh, tree diameter at breast height.

### Sampling and standardization of multi‐taxon biodiversity data

The taxonomic groups included in this study were vascular plants, birds, saproxylic fungi, and saproxylic beetles, all with information at the species level. These groups represent primary producers, consumers of different stages in the food web, and decomposers in forest ecosystems. Vascular plants were surveyed using rectangular and circular subplots of different sizes. Birds were detected visually and acoustically through the point‐count method. Saproxylic fungi were sampled on deadwood pieces of different diameter thresholds (1, 7, and 10 cm) within the plots. The saproxylic beetles were collected using different numbers of window and/or emergence traps on deadwood pieces per plot (Burrascano et al., 2021).

Since the sampling effort varied among sites (Appendix S1: Tables S4–S7), it was necessary to standardize the species richness values. We estimated the expected species richness per site from our sample‐based incidence data, using the “estimateD” function from the “iNEXT” package (Hsieh et al., 2016). We set the sampling completeness to 95%, a threshold often used in biodiversity studies (Grey et al., 2018; Monleon‐Getino & Frias‐Lopez, 2020; Nikkeshi et al., 2021). The standardized species richness for all taxonomic groups used in the analysis was calculated as the ratio between the observed species richness per taxonomic group and plot and the expected species richness per site.

### Statistical analysis

**FIGURE 2:**
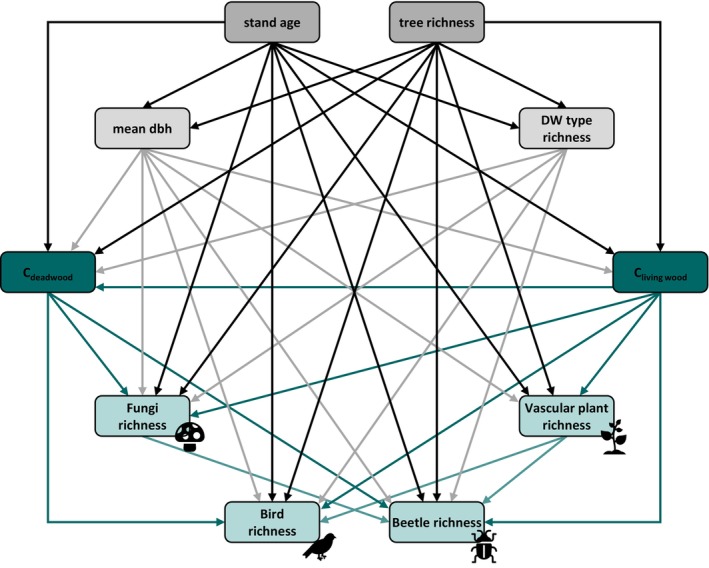
Expected ecological relationships between the variables considered in this study. For clarity, we omitted the boxes of mean temperature and precipitation and the arrows between mean temperature and precipitation and all other variables except stand age and tree richness. C, carbon; dbh, tree diameter at breast height; DW, deadwood. A table with all considered connections between the predictor and response variables is provided in Appendix S1: Table S7. Figure created by the authors using icons of fungi, birds, beetles, and plants from Microsoft PowerPoint under a Microsoft 365 license.

The data (Lunow et al., 2026) were analyzed using R 4.3.1 (R Core Team, 2023). To understand the relationship between forest structural characteristics, carbon stocks, and the richness of different taxonomic groups, we used structural equation models (SEM). With SEMs, we can understand complex ecological relationships between the predictor and response variables. As a first step, we set up a conceptual model of the connections between the response and predictor variables based on the available ecological literature (Figure 2, Appendix S1: Table S8). We calculated eight linear mixed‐effects models for each of the hypothesized connections in the conceptual model, with site (factor with 19 levels), forest type (factor with 5 levels), and silvicultural management (factor with 4 levels) as random effects. After the carbon stocks of deadwood and living wood were log‐transformed, the requirements of normal distribution and variance homogeneity of the linear mixed‐effects model residuals were met. The eight linear mixed‐effects models and the interrelation of mean temperature and annual precipitation (as a correction error) were fit with the “psem” function from the “piecewiseSEM” package (Lefcheck, 2016). This approach estimates path coefficients for each model separately and evaluates the overall model fit using tests of directed separation (Lefcheck, 2016). As all variables were scaled to mean = 0 and standard deviation = 1, the path coefficients within the model can be directly compared. To validate the SEM and to estimate the indirect, total, and mediator effects in addition to the direct effects within the model, we calculated a sampling distribution using the “semBoot” function (nboot = 999) included in the “semEff” package (Murphy, 2022).

## RESULTS

**FIGURE 3:**
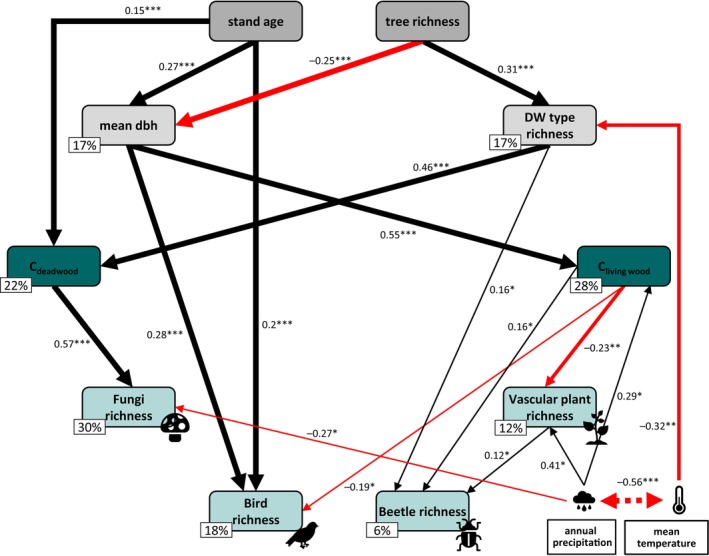
Path model (Fisher's *C* = 23.183, *p* = 0.057; note that *p* values >0.05 indicate statistical support) showing the complex relationship of forest structural characteristics, carbon stocks and the richness of different taxonomic groups. The numbers next to the arrows represent the standardized path coefficients (***p < 0.001, **p < 0.01, *p < 0.05). The thickness of the arrows is scaling with the significance values, while arrow color represents positive (black) and negative (red) relationships. The dashed line between mean temperature and annual precipitation depicts the error correction between these two variables. The percentage values indicate the marginal *R* ^2^ of each of the response variables. For clarity, we have omitted the non‐significant arrows and path coefficients in the figure. C, carbon; dbh, tree diameter at breast height; DW, deadwood. Full details of the models are provided in Appendix S1: Table S9. Figure created by the authors using icons of fungi, bird, beetle, plant, cloud and thermometer from Microsoft PowerPoint under a Microsoft 365 license.

The structural equation model resulted in complex relationships between the forest structural characteristics, carbon stocks, the richness of different taxonomic groups, and the site‐specific climatic conditions (Figure 3, Appendix S1: Table S9). The model was statistically reliable (Fisher's *C* = 23.183, *p* = 0.057; note that *p* values >0.05 indicate statistical support). One of the underlying predictors in the model, stand age, positively correlated with mean tree dbh, carbon stock in deadwood and bird species richness. Deadwood type richness was positively related to the other underlying predictor of the model, tree species richness, while mean tree dbh was negatively related to tree species richness. Mean tree dbh in turn was positively correlated with carbon in living wood and bird species richness. While carbon in living wood increased with increasing mean tree dbh, carbon stocks in deadwood increased with increasing stand age and deadwood type richness. The statistical effect of deadwood type richness on the amount of carbon in deadwood was even more than three times as high as its statistical effect on bird species richness. Saproxylic beetle species richness was positively correlated with the diversity of deadwood. Furthermore, carbon in deadwood was positively related to the species richness of saproxylic fungi, while carbon in living wood statistically affected saproxylic beetle richness positively, and bird and vascular plant species richness negatively. The model showed no direct effects of any forest structural characteristic on the species richness of saproxylic fungi and vascular plants. Saproxylic beetles were positively correlated with the species richness of vascular plants. Deadwood type richness was related to average temperature with higher temperatures coinciding with a decrease in deadwood type richness. With higher annual precipitation, vascular plant species richness and carbon in living wood increased but saproxylic fungi species richness decreased.

**FIGURE 4:**
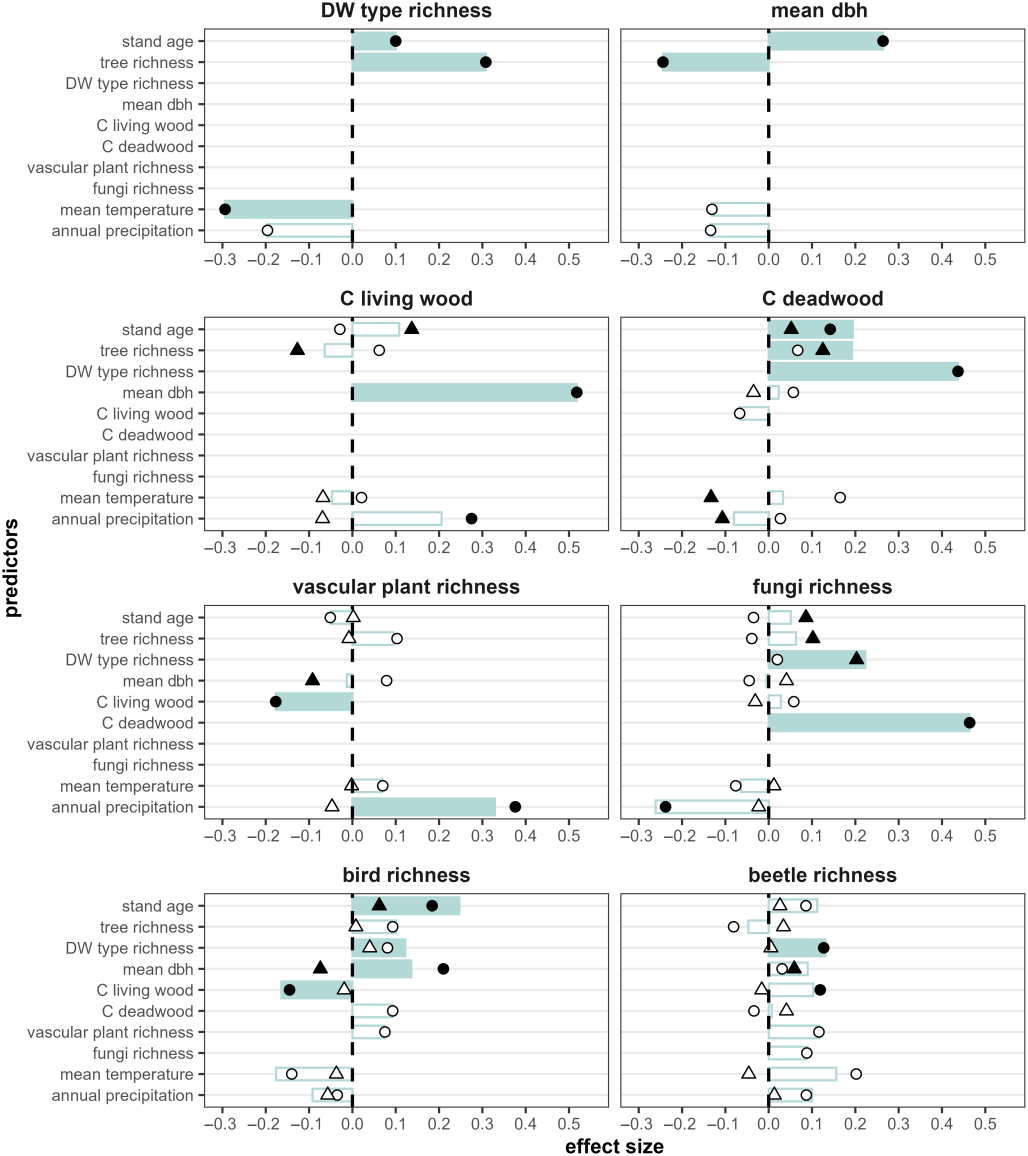
Bootstrapped direct (points) and indirect (triangles) effects of the different predictors on the eight response variables. Black filling of the points and triangles indicate significant direct and indirect effects. Turquoise boxes show the total effects of the predictors on the response variables. Filled boxes indicate significant total effects. Missing signs and bars mean that this effect was not considered in our conceptual model. C, carbon; dbh, tree diameter at breast height; DW, deadwood. Detailed table of the effects is provided in Lunow, 2026.

Besides those direct effects, we also detected significant indirect and total effects (Figure 4; Lunow, 2026). The richness of tree species per plot and stand age had positive total statistical effects on deadwood type richness, while increasing mean temperature in total led to a decrease in deadwood type richness. The statistical effects of tree species richness and stand age on mean tree dbh had almost the same strength, but were of opposite sign, respectively negative and positive. Carbon stocks in living wood and in deadwood were mainly indirectly related to tree species richness and stand age. Carbon in living wood was directly correlated with annual precipitation, while the correlation of deadwood carbon and annual precipitation was negative and indirect. In addition to that, mean temperature had a negative indirect relationship to carbon stocks in deadwood. Despite indirect statistical effects of stand age and tree species richness on carbon stocks in living and dead wood, the bootstrapped model did not identify mean tree dbh as a significant mediator (Lunow, 2026), likely due to other underlying mechanisms not accounted for in this study. Overall, carbon stocks in living wood were mainly related to mean tree dbh, while deadwood carbon stocks were related to deadwood type richness. The carbon stocks in living wood per plot were significantly mediating the indirect statistical effects of the underlying predictors on vascular plant and bird species richness, while carbon in deadwood was mediating the positive indirect statistical effects of stand age, tree richness, and deadwood type richness on the species richness of saproxylic fungi. Considering the total statistical effects, increasing deadwood type richness led to a higher species richness in saproxylic fungi, birds, and saproxylic beetles and to an increase in carbon stocks in deadwood.

## DISCUSSION

Based on our results, the structural characteristics of forests play a key role for carbon storage and the richness of different taxonomic groups. Both biodiversity and the carbon stocks in living and dead wood were especially correlated with mean tree dbh and the diversity of deadwood. In turn, both carbon stocks mediated additional statistical effects of forest structural characteristics on the species richness of different taxonomic groups.

### Forest structural characteristics directly influence living and dead wood carbon stocks and multi‐taxon species richness

Our results show indirect statistical effects of stand age (positive) and tree richness (negative) on carbon stocks in living wood, with mean tree dbh marginally significantly mediating these effects. Upon disentangling the individual links, we found that the mean dbh of trees increased in older forest stands, as reported in other studies (Rohner et al., 2013). Furthermore, we observed that higher tree species richness was associated with lower mean dbh, and consequently, lower living tree carbon stocks. This finding is consistent with recent literature (Lutz et al., 2012; Pretzsch & Schütze, 2014), as trees with a high dbh play a pivotal role in forest carbon storage (Lutz et al., 2018; Mildrexler et al., 2020).

Our findings underline that a higher stand age directly and indirectly leads to a higher amount of carbon stored in deadwood, in line with previous findings (Bujoczek et al., 2024). This is due to the progressive accumulation of deadwood in older forests, especially in those with non‐intense management (Bujoczek et al., 2024; Sturtevant et al., 1997). Accordingly, our results indicate an increase in deadwood type richness with stand age in temperate European managed forests. Including decay classes of deadwood in the calculation of deadwood type richness, we expected and found that an increase in tree species richness leads to an increase in deadwood type richness due to varying decay rates of different tree species (Kahl et al., 2017). Additionally, we found that stand age and tree species richness influence deadwood carbon stocks and type richness. This association is likely expressed through the shaping of microclimatic conditions at the level of the stand, thereby influencing the decomposition processes of deadwood (Wu et al., 2023) and the formation of a higher diversity of deadwood structures in older forests (Bujoczek et al., 2024).

Based on the possible mechanism that a higher tree species richness diversifies the canopy opening and therefore leads to a diverse light intensity in the understory (Barbier et al., 2008; but see Burrascano et al., 2018; Dormann et al., 2020), we expected the species richness of trees and understory vascular plants to be related. However, notwithstanding the fact that tree species richness values in our study covered the whole range of tree species richness in temperate European forests (i.e., ranged from one in monocultures to highly mixed forests with 14 tree species at the northern Swabian Alb in Germany), we found this relation to be only marginally significant, raising uncertainties on the overstory/understory link in terms of vascular plant species richness.

Our results support the findings of other studies that older forests, larger trees, and a higher diversity of deadwood increase the bird species richness due to a greater likelihood of tree cavities and better food resources, besides larger amounts of deadwood (Basile et al., 2021; Larrieu et al., 2019; Moning & Müller, 2009). Possibly, the indirect positive relationship of stand age on bird species richness is explained through a higher occurrence of microhabitats in older forests with larger trees (Paillet et al., 2018; Rohner et al., 2013), although we found no significant mediator effect in our analysis.

We found that a higher deadwood type richness resulted in higher species richness values for different taxonomic groups. As a matter of fact, different species of saproxylic beetles depend on different diameter sizes and decay classes of deadwood (Andringa et al., 2019; Heidrich et al., 2020; Seibold et al., 2016). Our results also show a positive effect of an increasing vascular plant richness on the species richness of saproxylic beetles, which can be explained through the provision of food resources to adults through flowering plants, in line with previous findings (Edelmann et al., 2022; Heidrich et al., 2020; Penone et al., 2019).

### Living and dead wood carbon stocks have diverse links to the species richness of multiple taxonomic groups

We found that vascular plant species richness decreased with an increasing amount of carbon stocks in living wood and, indirectly, with a higher mean tree dbh. Light and its heterogeneity are important factors driving vascular plant diversity in forests (Dormann et al., 2020; Helbach et al., 2022; Márialigeti et al., 2016; Tinya & Ódor, 2016). Our data show that forests with higher carbon stocks are beech forests with a time since the last intervention of more than 50 years. The dense canopy of these forests prevents higher light intensities from reaching the forest floor and hinders the formation of heterogeneous light conditions. Additionally, as our analysis was limited to even‐aged forests, there may be a lack of vertical and horizontal structural variability in forests with higher carbon stocks, leading to more homogeneous light conditions on the forest floor (Davies & Asner, 2014; De Frenne et al., 2019; Heidrich et al., 2020). Therefore, we assume that the decrease in plant species richness is particularly related to a decrease in non‐forest plant species that are not adapted to lower light intensities in the understory (Sabatini et al., 2014, 2019) and homogeneous light conditions that inhibit the development of a diverse vascular plant community in the understory (Helbach et al., 2022; Tinya & Ódor, 2016).

Previous studies have shown that fungi species diversity in forests is driven by stand age and the amount of deadwood (Doerfler et al., 2018; Zeller et al., 2023). Here, we found that increasing stand age had this positive relationship with saproxylic fungi species richness in an indirect way through its influence on the carbon stocks in deadwood. The carbon stock in deadwood played an important role as it acted as a mediator for the indirect positive relationships of stand age and deadwood type richness with fungi species richness, likely through the increased niche availability and specialization of saproxylic fungi (Rieker et al., 2022). Increasing the amount of deadwood leads to a higher resource availability and a higher number of niches, which can be used by saproxylic fungi species (Doerfler et al., 2018). This explains the strong relationship of deadwood carbon stocks and the richness of saproxylic fungi our results show. Previous studies also showed that birds profit from higher living carbon stocks (Hatanaka et al., 2011; Lecina‐Diaz et al., 2018; Sabatini et al., 2019). By contrast, we found that bird species richness was negatively correlated with carbon stocks in living wood and this carbon stock mediated the negative indirect relationship of mean tree dbh on bird species richness. As our study only included even‐aged forests, the forests analyzed here may consist of trees of a similar size with high accumulated biomass and a low structural diversity. Consequently, these forests appear to lack the essential resources required by certain bird species belonging to specific nesting guilds (e.g., hollow‐nesting birds) (Hanle et al., 2020). Additionally, forests with high levels of carbon stocks in living wood can lack in ecological complex carbon, that is, carbon in structures that favor forest species such as wide‐branching canopies, shrubs, bare ground, and large, hollow‐bearing, and dead standing trees (Hatanaka et al., 2011). The diverging correlations between mean tree dbh and carbon stocks in living wood and bird species richness reflect the fact that mean tree dbh in this study represent bird habitat quality, whereas carbon stocks in living wood only represent biomass accumulation, regardless of bird habitat quality.

Our results show an increase in saproxylic beetle species richness with an increase in the carbon stocks of living wood, likely linked to the fact that a higher overall biomass in a forest ecosystem leads to a diversification of saproxylic organisms (Parisi et al., 2019). While other studies found a positive effect of the amount of deadwood on the diversity of saproxylic beetles (Bouget et al., 2013; Doerfler et al., 2018; Sandström et al., 2019), we found no such effect. Based on this, our results support the findings of Lassauce et al. (2011) that a high richness of deadwood types is important for saproxylic beetles.

The analyzed forest structures, carbon stocks, and taxonomic species richness are partly correlated with the site‐specific climatic conditions (mean annual temperature, annual precipitation). The negative correlations of temperature and precipitation and deadwood type richness can be attributed to increased decomposer activity and subsequent accelerated deadwood decomposition in warmer, wetter conditions (Conant et al., 2011; Edman et al., 2021). The positive correlation between higher carbon stocks in living wood and precipitation is explained by higher tree growth rates in areas with higher precipitation (Harvey et al., 2020). Although none of the sites considered in this study experience substantial water stress, we found that the richness of vascular plants increased with increasing precipitation, in line with experimental findings, albeit with a stronger effect on local scales (Korell et al., 2021). Taking a broader view, this result is also consistent with another study, which showed that the taxonomic diversity of plants increased due to higher precipitation, based on the global climate‐richness pattern (Harrison et al., 2020). The negative correlation of saproxylic fungi richness and precipitation in our study confirms that fungal communities change due to macroclimatic conditions (Heilmann‐Clausen et al., 2014; Thorn et al., 2018). Specifically, Thorn et al., (2018) demonstrate that, as elevation increases (i.e., as precipitation increases and temperature decreases), the taxonomic diversity of saproxylic fungi decreases. Our results support this finding, demonstrating a negative correlation between saproxylic fungi and precipitation, as well as a negative relationship between temperature and precipitation.

### Study limitations and opportunities

Analyzing a large dataset deriving from various projects, we faced different limitations. Firstly, our analysis is limited to the five forest types that were in the focus of such projects. Secondly, due to the different sampling approaches, we had to limit the set of taxonomic groups and forest structural characteristics based on data availability and harmonization possibilities. Finally, the analyzed data only relate to even‐aged forests because in uneven‐aged forests, management interventions do not affect the whole stand and therefore the time since last intervention (which we used to define stand age) would not be directly comparable to even‐aged forests. Additionally, considering a broader temporal scale of natural forest succession could reveal more positive correlations between forest structures, carbon storage, and biodiversity (Mikoláš et al., 2021). However, temporal modeling is currently the only way to study such effects, due to the absence of older stages of forest succession after management in temperate Europe.

Nevertheless, the opportunity of modeling forest relationships across a wide extent in Europe accounting for the plot scale mechanisms led to highly relevant results. We identified two variables that are crucial for the ecology and management of temperate European forests: Deadwood type richness plays a major role in the process of storing carbon in deadwood and enhancing the richness of taxonomic groups from different hierarchical orders. Living carbon stocks act as a suppressor for vascular plant and bird species richness and as a supporter for saproxylic beetle species richness. Importantly, our analysis suggests that the indirect impact of stand structural characteristics on species richness across multiple taxa is statistically mediated by carbon stocks. However, this relationship may be partly influenced by the characteristics of the selected even‐aged stands. The latter displayed a diverse range of relationships both with forest structural characteristics and, more importantly, the diversity of organisms in forest ecosystems.

By considering other diversity measures (e.g., abundance, Shannon‐diversity, or beta‐diversity), belowground carbon, further forest structural characteristics, and nonlinear relationships among these variables, future studies may further disentangle the interplay of biodiversity, carbon, and forest structural characteristics.

## CONCLUSION

Overall, our findings support the idea that achieving forest multifunctionality (e.g., high carbon stocks and biodiversity) requires consideration of the complex links between forest functions, structural characteristics, and species diversity that can be targeted according to management objectives. Current sustainable forest management strategies focus on an increase of wood biomass in order to increase carbon storage and biodiversity. This is either achieved through extended rotational periods, that is, a broader stand age range, or by promoting the occurrence of large individual trees. While these strategies may have positive effects on the species richness of some taxonomic groups, other groups will experience significant trade‐offs or need the promotion of other structural features, that is, deadwood. Therefore, current policies are not effectively targeting both the climate and biodiversity crises but could instead benefit from increasing deadwood diversity.

## AUTHOR CONTRIBUTIONS

Julian Lunow, Sabina Burrascano, Lorenzo Balducci, and Nadja K. Simons conceived the ideas and designed the methodology. Julian Lunow analyzed the data. Julian Lunow wrote the manuscript and the appendix with the support of Sabina Burrascano and Nadja K. Simons. The data were collected and/or managed by Sabina Burrascano, Lorenzo Balducci, Francesco Chianucci, Lucas Chojnacki, Inken Doerfler, Jeňýk Hofmeister, Jan Hošek, Péter Ódor, Peter Schall, and Tommaso Sitzia. All authors contributed critically to the drafts.

## CONFLICT OF INTEREST STATEMENT

The authors declare no conflict of interest.

## Supporting information


Appendix S1.

## Data Availability

Data and code (Lunow et al., 2026) are available in Zenodo at https://doi.org/10.5281/zenodo.18254755. Additional data (Lunow, 2026) are available in Figshare at https://doi.org/10.6084/m9.figshare.27118065. This work utilized data that are publicly available in the Biodiversity Exploratories Information System (BExIS) repository as follows: dataset ID 16866 (Goßner et al., 2013); dataset ID 18547 (Fischer, 2014); dataset ID 21448 (Tschapka et al., 2017); dataset ID 23686 (Fischer et al., 2018); dataset ID 24546 (Schall et al., 2019); and dataset ID 31718 (Schall, 2024).
