# Supplementary material for: Structural characteristics mediate forest mitigation potential against climate change and biodiversity loss
Source: Ecol Appl. 2026 Mar 16;36(2):e70211. doi: 10.1002/eap.70211 (PMC12991856; doi:10.1002/eap.70211)
Supplement: Supplementary file 1 — Appendix S1. [file EAP-36-e70211-s001.pdf]

Appendix S1

Structural characteristics mediate forest mitigation potential against climate change and biodiversity loss

Julian Lunow, Sabina Burrascano, Lorenzo Balducci, Francesco Chianucci, Lucas Chojnacki, Inken Doerfler, Jeňýk Hofmeister, Jan Hošek, Péter Ódor, Peter Schall, Tommaso Sitzia, Nadja K. Simons

Ecological Applications

Table S1: Minimum and maximum temperature and precipitation of the different plots per forest site. The values are based on the climate period of 1981-2010. The data on temperature and precipitation was retrieved from the Chelsa-Climate database with a resolution of ~1 km (Karger et al. 2017). *The first two letters from the siteID indicate the country.*

Site	temperature min (°C)	temperature max (°C)	annual precipitation min (mm)	annual precipitation max (mm)
CZ_JH2_L1	7.45	8.15	626.6	666.6
CZ_JH2_L2	6.35	7.05	879.6	897
CZ_JH2_L3	5.95	6.65	1037	1088.6
CZ_JH2_L4	6.95	7.35	681.3	750.7
CZ_JH2_L5	8.95	9.15	666.2	689.2
CZ_JH2_L6	9.65	9.75	557.1	560.3
DE_ID	8.25	8.75	797	840.9
DE_PS_Alb_N	7.15	7.85	896.2	1013.8
DE_PS_Alb_S	7.05	7.75	911.7	959.7
DE_PS_Alb_W	7.15	7.65	914.2	971.6
DE_PS_Hai_N	7.25	7.85	725.2	869.2
DE_PS_Hai_S	7.65	8.45	695.5	864.9
DE_PS_Sch_N	8.55	8.95	579.5	602.2
DE_PS_Sch_S	8.75	9.05	579.9	633.6
IT_Cilento	8.45	8.55	1035.8	1080
IT_GranSasso	6.15	8.55	1150.6	1195.1

Table S2: Number of plots per country, site, forest category and silvicultural management. The different forest categories are: category 2: hemiboreal forest and nemoral coniferous and mixed broadleaved-coniferous forest, category 5: mesophytic deciduous forest, category 6: beech forest, category 7: mountainous beech forest, category 14: plantations and self-sown exotic forests.

Country	Site	forest category	silvicultural management	count
Czech Republic	CZ_JH1_L1	5	retention clearcutting	1
Czech Republic	CZ_JH1_L1	6	simple clearcutting	5
Czech Republic	CZ_JH1_L1	14	simple clearcutting	8
Czech Republic	CZ_JH1_L2	7	retention clearcutting	2
Czech Republic	CZ_JH1_L2	7	simple clearcutting	6
Czech Republic	CZ_JH1_L2	14	retention clearcutting	1
Czech Republic	CZ_JH1_L2	14	simple clearcutting	5
Czech Republic	CZ_JH1_L3	7	retention clearcutting	1

Czech Republic	CZ_JH1_L3	7	simple clearcutting	6
Czech Republic	CZ_JH1_L3	14	retention clearcutting	2
Czech Republic	CZ_JH1_L3	14	simple clearcutting	5
Czech Republic	CZ_JH1_L4	5	simple clearcutting	1
Czech Republic	CZ_JH1_L4	6	simple clearcutting	3
Czech Republic	CZ_JH1_L4	7	retention clearcutting	2
Czech Republic	CZ_JH1_L4	14	retention clearcutting	2
Czech Republic	CZ_JH1_L4	14	simple clearcutting	8
Czech Republic	CZ_JH1_L5	5	retention clearcutting	2
Czech Republic	CZ_JH1_L5	5	simple clearcutting	2
Czech Republic	CZ_JH1_L5	6	simple clearcutting	1
Czech Republic	CZ_JH1_L5	14	simple clearcutting	6
Czech Republic	CZ_JH1_L6	5	retention clearcutting	3
Czech Republic	CZ_JH1_L6	5	simple clearcutting	3
Czech Republic	CZ_JH1_L6	14	simple clearcutting	4
Germany	DE_ID_1	6	shelterwood	49
Germany	DE_ID_2	6	shelterwood	29
Germany	DE_PS_ALB_N	2	shelterwood	2
Germany	DE_PS_ALB_N	7	shelterwood	10
Germany	DE_PS_ALB_S	2	shelterwood	8
Germany	DE_PS_ALB_S	7	shelterwood	20
Germany	DE_PS_ALB_W	2	shelterwood	2
Germany	DE_PS_ALB_W	7	shelterwood	8
Germany	DE_PS_HAI_N	2	retention clearcutting	2
Germany	DE_PS_HAI_N	6	shelterwood	13
Germany	DE_PS_HAI_S	2	retention clearcutting	2
Germany	DE_PS_HAI_S	6	selection cutting	10
Germany	DE_PS_HAI_S	6	shelterwood	10
Germany	DE_PS_SCH_N	2	retention clearcutting	5
Germany	DE_PS_SCH_N	6	shelterwood	9
Germany	DE_PS_SCH_S	2	retention clearcutting	8
Germany	DE_PS_SCH_S	6	shelterwood	12
Italy	Cilento_N_1	7	shelterwood	5
Italy	Cilento_N_2	7	shelterwood	3
Italy	Gran_Sasso_1	7	shelterwood	10
Italy	Gran_Sasso_2	7	shelterwood	5

Table S3: Deadwood basic density (kgm^{-3}) for coarse woody debris, standing dead trees and stumps by group (conifers and broadleaves) and class of decay (1-5). Changed according to (Di Cosmo et al. 2013).

Decay class	Coarse woody debris		Standing dead trees		Stumps	
	**Conifers**	**Broadleaves**	**Conifers**	**Broadleaves**	**Conifers**	**Broadleaves**
1	411.5	513.0	442.9	522.8	495.5	511.3
2	389.8	468.6	423.9	525.8	450.9	494.3
3	342.0	444.3	384.3	513.7	397.9	451.0
4	295.4	344.0	354.4	438.6	344.7	410.4
5	265.9	255.1	372.3		271.2	306.8

Table S4: Sample area per site for vascular plants. Yes or No in the last column indicates if the vascular plant survey was split into subsamples per plot.

Site	sample area	split into subsamples
CZ_JH1_L1	55	YES
CZ_JH1_L2	55	YES
CZ_JH1_L3	55	YES
CZ_JH1_L4	55	YES
CZ_JH1_L5	55	YES
CZ_JH1_L6	55	YES
DE_ID	196	NO
DE_PS_ALB_N	400	YES
DE_PS_ALB_S	400	YES
DE_PS_ALB_W	400	YES
DE_PS_HAI_N	400	YES
DE_PS_HAI_S	400	YES
DE_PS_SCH_N	400	YES
DE_PS_SCH_S	400	YES
Cilento_N	1256	NO
Gran_Sasso	1256	NO

Table S5: Sample area for saproxylic fungi per site. Deadwood diameter indicates the diameter threshold for deadwood pieces on each plot. Yes or No in the last column indicates if the fungi survey was split into subsamples per plot.

Site	sample area	deadwood diameter	Nested
CZ_JH1_L1	2500	1	YES
CZ_JH1_L2	2500	1	YES
CZ_JH1_L3	2500	1	YES
CZ_JH1_L4	2500	1	YES
CZ_JH1_L5	2500	1	YES
CZ_JH1_L6	2500	1	YES
DE_ID	452.4	1	NO
DE_PS_ALB_N	400	7	YES
DE_PS_ALB_S	400	7	YES
DE_PS_ALB_W	400	7	YES
DE_PS_HAI_N	400	7	YES

DE_PS_HAI_S	400	7	YES
DE_PS_SCH_N	400	7	YES
DE_PS_SCH_S	400	7	YES
Cilento_N	530	10	NO
Gran_Sasso	530	10	NO

Table S6: Overall sampling time in minutes for birds per site. Yes or No in the last column indicates if the bird survey was repeated several times or not.

Site	sample time [min]	repeated
CZ_JH1_L1	25	YES
CZ_JH1_L2	25	YES
CZ_JH1_L3	25	YES
CZ_JH1_L4	25	YES
CZ_JH1_L5	25	YES
CZ_JH1_L6	25	YES
DE_ID	35	YES
DE_PS_ALB_N	300	YES
DE_PS_ALB_S	300	YES
DE_PS_ALB_W	300	YES
DE_PS_HAI_N	300	YES
DE_PS_HAI_S	300	YES
DE_PS_SCH_N	300	YES
DE_PS_SCH_S	300	YES
Cilento_N	450	YES
Gran_Sasso	450	YES

Table S7: Number of window and emergence traps in the different surveys for saproxylic beetles per site.

Site	number of window	number of emergence
CZ_JH1_L1	4	0
CZ_JH1_L2	4	0
CZ_JH1_L3	4	0
CZ_JH1_L4	4	0
CZ_JH1_L5	4	0
CZ_JH1_L6	4	0
DE_ID	3	0
DE_PS_ALB_N	4	0
DE_PS_ALB_S	4	0
DE_PS_ALB_W	4	0
DE_PS_HAI_N	4	0
DE_PS_HAI_S	4	0
DE_PS_SCH_N	4	0
DE_PS_SCH_S	4	0
Cilento_N	1	3
Gran_Sasso	1	3

Table S8: Overview of the eight response variables and their predictors used to set up the piecewise SEM. The expected ecological relationships were selected based on the cited literature.

Response	Predictors	Reference
**mean DBH**	stand age	Lee et al. (2024); Rohner, Bugmann, and Bigler (2013)
	tree richness	Pretzsch and Schütze (2016; 2014)
	mean temperature	Harvey et al. (2020)
	annual precipitation	Harvey et al. (2020)
**DW type richness**	stand age	Bujoczek, Bujoczek, and Zięba (2024)
	tree richness	Herrmann, Kahl, and Bauhus (2015)
	mean temperature	Edman, Hagos, and Carlsson (2021); Kahl et al. (2017)
	annual precipitation	Kahl et al. (2017)
**C living wood**	stand age	Lasky et al. (2014); Pretzsch and Hilmers (2024)
	tree richness	Cavanaugh et al. (2014); Sullivan et al. (2017)
	mean DBH	Lutz et al. (2018; 2012); Mildrexler et al. (2020)
	mean temperature	Harvey et al. (2020)
	annual precipitation	Harvey et al. (2020)
**C deadwood**	stand age	Sturtevant et al. (1997)
	tree richness	Wu et al. (2023)
	mean DBH	McGee, Leopold, and Nyland (1999); Oettel et al. (2020)
	DW type richness	Harmon et al. (2004)
	C living	Oettel et al. (2020)
	mean temperature	Harmon et al. (2004); Oettel et al. (2020)
	annual precipitation	Harmon et al. (2004)
**Vascular plant**	stand age	Hilmers et al. (2018); Zeller et al. (2023)
**richness**	tree richness	Larrieu et al. (2019); Tinya et al. (2021)
	mean DBH	Burrascano et al. (2018); Chamagne et al. (2016)
	C living wood	Sabatini et al. (2019)
	mean temperature	Govaert et al. (2021); Zellweger et al. (2016)
	annual precipitation	Otsu et al. (2023)
**Bird richness**	stand age	Zeller et al. (2023)
	tree richness	Zeller et al. (2023)
	mean DBH	Benedetti et al. (2021); Tinya et al. (2021)
	DW type richness	Larrieu et al. (2019)
	C living wood	Sabatini et al. (2019)
	C deadwood	Bütler et al. (2004); Lohr, Gauthreaux, and Kilgo (2002)
	Vascular plant richness	Dagan and Izhaki (2019); Lynch and Whigham (1984)
	mean temperature	Zhang, Kissling, and He (2013)
	annual precipitation	Zhang, Kissling, and He (2013)
**Fungi richness**	stand age	Hilmers et al. (2018); Zeller et al. (2023)
	tree richness	Heine et al. (2019); Rieker et al. (2022)
	mean DBH	Tinya et al. (2021)
	DW type richness	Hoppe et al. (2016); Lassauce et al. (2011)
	C living wood	Sabatini et al. (2019)
	C deadwood	Doerfler et al. (2018); Sandström et al. (2019)

	mean temperature	Thorn et al. (2018); Tinya et al. (2021)
	annual precipitation	Thorn et al. (2018)
**Beetle richness**	stand age	Hilmers et al. (2018); Larrieu et al. (2019)
	tree richness	Chamagne et al. (2016); Larrieu et al. (2019)
	mean DBH	Larrieu et al. (2019); Rappa et al. (2022)
	DW type richness	Heidrich et al. (2020); Larrieu et al. (2019); Lassauce et al. (2011)
	C living wood	Parisi et al. (2019)
	C deadwood	Doerfler et al. (2018); Gossner et al. (2013); Sandström et al. (2019)
	Vascular plant richness	Edelmann et al. (2022); Heidrich et al. (2020)
	Fungi richness	Bouget, Larrieu, and Brin (2014); Økland et al. (1996)
	mean temperature	Müller et al. (2015)
	annual precipitation	Thorn et al. (2018)

Table S9: Direct effects of the different predictor variables on the response variables in the structural equation model. C = carbon, DBH = diameter at breast height, DW = deadwood.

Response	Predictor	Estimate	StdErr	DF	Crit.Value	p-value	Sign.
DW type richness	tree richness	0.313	0.061	229.339	23.916	< 0.001	***
DW type richness	stand age	0.101	0.052	275.431	3.592	0.059	
DW type richness	mean temperature	-0.323	0.092	41.061	10.525	0.002	**
DW type richness	annual precipitation	-0.217	0.104	16.654	2.979	0.103	
mean DBH	tree richness	-0.249	0.063	136.468	13.829	< 0.001	***
mean DBH	stand age	0.266	0.054	167.742	20.948	< 0.001	***
mean DBH	mean temperature	-0.152	0.086	36.803	2.638	0.113	
mean DBH	annual precipitation	-0.157	0.095	24.857	1.733	0.200	
C living	tree richness	0.064	0.048	272.470	1.665	0.198	
C living	stand age	-0.030	0.038	196.530	0.580	0.447	
C living	mean DBH	0.546	0.039	278.348	195.748	< 0.001	***
C living	mean temperature	0.023	0.093	177.042	0.055	0.814	
C living	annual precipitation	0.294	0.126	43.944	4.768	0.034	*
C deadwood	tree richness	0.070	0.049	288.309	1.968	0.162	
C deadwood	stand age	0.151	0.039	268.276	14.267	< 0.001	***
C deadwood	mean DBH	0.077	0.050	288.744	2.348	0.127	
C deadwood	DW type richness	0.458	0.043	284.261	110.212	< 0.001	***
C deadwood	C living	-0.088	0.058	289.805	2.183	0.141	
C deadwood	mean temperature	0.178	0.092	168.218	3.556	0.061	
C deadwood	annual precipitation	0.029	0.127	50.917	0.045	0.833	
Vascular plant richness	tree richness	0.106	0.061	290.346	2.867	0.092	
Vascular plant richness	stand age	-0.054	0.050	276.650	1.102	0.295	
Vascular plant richness	mean DBH	0.105	0.063	285.094	2.620	0.107	
Vascular plant richness	C living	-0.229	0.073	259.661	9.003	0.003	**
Vascular plant richness	mean temperature	0.076	0.111	109.105	0.437	0.510	
Vascular plant richness	annual precipitation	0.411	0.142	34.890	6.867	0.013	*
Fungi richness	tree richness	-0.042	0.060	279.595	0.459	0.499	
Fungi richness	stand age	-0.038	0.050	277.064	0.538	0.464	
Fungi richness	mean DBH	-0.059	0.060	255.445	0.892	0.346	

Fungi richness	DW type richness	0.025	0.062	286.815	0.153	0.696	
Fungi richness	C deadwood	0.567	0.070	209.291	58.690	< 0.001	***
Fungi richness	C living	0.074	0.069	185.164	1.021	0.314	
Fungi richness	mean temperature	-0.086	0.094	62.156	0.742	0.392	
Fungi richness	annual precipitation	-0.268	0.111	24.147	4.347	0.048	*
Bird richness	tree richness	0.099	0.062	261.870	2.231	0.137	
Bird richness	stand age	0.201	0.050	260.049	14.598	< 0.001	***
Bird richness	mean DBH	0.283	0.063	264.936	18.569	< 0.001	***
Bird richness	DW type richness	0.101	0.064	283.164	2.327	0.128	
Bird richness	C deadwood	0.116	0.073	263.611	2.282	0.132	
Bird richness	C living	-0.192	0.074	214.091	5.894	0.016	*
Bird richness	Vascular plant richness	0.078	0.056	219.869	1.712	0.192	
Bird richness	mean temperature	-0.152	0.112	116.866	1.700	0.195	
Bird richness	annual precipitation	-0.039	0.140	34.300	0.062	0.805	
Beetle richness	tree richness	-0.086	0.060	262.659	1.802	0.181	
Beetle richness	stand age	0.094	0.049	263.230	3.396	0.067	
Beetle richness	mean DBH	0.043	0.062	263.048	0.440	0.508	
Beetle richness	DW type richness	0.156	0.062	283.779	6.013	0.015	*
Beetle richness	C deadwood	-0.047	0.078	259.775	0.326	0.569	
Beetle richness	C living	0.159	0.073	211.782	4.210	0.041	*
Beetle richness	Vascular plant richness	0.120	0.055	237.143	4.356	0.038	*
Beetle richness	Fungi richness	0.101	0.057	253.471	2.906	0.090	
Beetle richness	mean temperature	0.219	0.113	145.953	3.518	0.063	
Beetle richness	annual precipitation	0.096	0.148	39.200	0.354	0.555	
~~mean temperature	~~annual precipitation	-0.556		299.000	-11.577	< 0.001	***

References

Benedetti, Yanina, Eleftherios Kapsalis, Federico Morelli, and Vassiliki Kati. 2021. ‘Sacred Oak Woods Increase Bird Diversity and Specialization: Links with the European Biodiversity Strategy for 2030’. *Journal of Environmental Management* 294: 112982. https://doi.org/10.1016/j.jenvman.2021.112982.

Bouget, Christophe, Laurent Larrieu, and Antoine Brin. 2014. ‘Key Features for Saproxylic Beetle Diversity Derived from Rapid Habitat Assessment in Temperate Forests’. *Ecological Indicators* 36: 656–64. https://doi.org/10.1016/j.ecolind.2013.09.031.

Bujoczek, Leszek, Małgorzata Bujoczek, and Stanisław Zięba. 2024. ‘How Do Stand Features Shape Deadwood Diversity?’ *Forest Ecology and Management* 553: 121609. https://doi.org/10.1016/j.foreco.2023.121609.

Burrascano, Sabina, Francesco Ripullone, Liliana Bernardo, Marco Borghetti, Emanuela Carli, Michele Colangelo, Carmen Gangale, et al. 2018. ‘It’s a Long Way to the Top: Plant Species Diversity in the Transition from Managed to Old-growth Forests’. *Journal of Vegetation Science* 29 (1): 98–109. https://doi.org/10.1111/jvs.12588.

Bütler, Rita, Per K. Angelstam, P. Ekelund, and Rodolphe Schlaepfer. 2004. ‘Dead Wood Threshold Values for the Three-Toed Woodpecker Presence in Boreal and Sub-Alpine Forest’. *Biological Conservation* 119 (3): 305–18. https://doi.org/10.1016/j.biocon.2003.11.014.

Cavanaugh, Kyle C., J. Stephen Gosnell, Samantha L. Davis, Jorge Ahumada, Patrick Boundja, David B. Clark, Badru Mugerwa, et al. 2014. ‘Carbon Storage in Tropical Forests Correlates with Taxonomic Diversity and Functional Dominance on a Global Scale: Biodiversity and Aboveground Carbon Storage’. *Global Ecology and Biogeography* 23 (5): 563–73. https://doi.org/10.1111/geb.12143.

Chamagne, Juliette, C. E. Timothy Paine, Donald R. Schoolmaster, Robert Stejskal, Daniel Volařík, Jan Šebesta, Filip Trnka, et al. 2016. ‘Do the Rich Get Richer? Varying Effects of Tree Species Identity and Diversity on the Richness of Understory Taxa’. *Ecology* 97 (9): 2364–73. https://doi.org/10.1002/ecy.1479.

Dagan, Uzi, and Ido Izhaki. 2019. ‘Understory Vegetation in Planted Pine Forests Governs Bird Community Composition and Diversity in the Eastern Mediterranean Region’. *Forest Ecosystems* 6 (1): 29. https://doi.org/10.1186/s40663-019-0186-y.

Di Cosmo, Lucio, Patrizia Gasparini, Alessandro Paletto, and Michaela Nocetti. 2013. ‘Deadwood Basic Density Values for National-Level Carbon Stock Estimates in Italy’. *Forest Ecology and Management* 295: 51–58. https://doi.org/10.1016/j.foreco.2013.01.010.

Doerfler, Inken, Martin M. Gossner, Jörg Müller, Sebastian Seibold, and Wolfgang W. Weisser. 2018. ‘Deadwood Enrichment Combining Integrative and Segregative Conservation Elements Enhances Biodiversity of Multiple Taxa in Managed Forests’. *Biological Conservation* 228: 70–78. https://doi.org/10.1016/j.biocon.2018.10.013.

Edelmann, Pascal, Didem Ambarlı, Martin M. Gossner, Peter Schall, Christian Ammer, Beate Wende, Ernst-Detlef Schulze, et al. 2022. ‘Forest Management Affects Saproxylic Beetles through Tree Species Composition and Canopy Cover’. *Forest Ecology and Management* 524: 120532. https://doi.org/10.1016/j.foreco.2022.120532.

Edman, Mattias, Saba Hagos, and Fredrik Carlsson. 2021. ‘Warming Effects on Wood Decomposition Depend on Fungal Assembly History’. *Journal of Ecology* 109 (4): 1919–30. https://doi.org/10.1111/1365-2745.13617.

Gossner, Martin M., Andreas Floren, Wolfgang W. Weisser, and Karl Eduard Linsenmair. 2013. ‘Effect of Dead Wood Enrichment in the Canopy and on the Forest Floor on Beetle Guild Composition’. *Forest Ecology and Management* 302: 404–13. https://doi.org/10.1016/j.foreco.2013.03.039.

Govaert, Sanne, Pieter Vangansbeke, Haben Blondeel, Kathy Steppe, Kris Verheyen, and Pieter De Frenne. 2021. ‘Rapid Thermophilization of Understorey Plant Communities in a 9 Year-long Temperate Forest Experiment’. *Journal of Ecology* 109 (6): 2434–47. https://doi.org/10.1111/1365-2745.13653.

Harmon, Mark E., Jerry F. Franklin, Frederick J. Swanson, Phillip Sollins, Stanley V. Gregory, John D. Lattin, N.H. Anderson, et al. 2004. ‘Ecology of Coarse Woody Debris in Temperate Ecosystems’. In *Advances in Ecological Research*, vol. 34. Elsevier. https://doi.org/10.1016/S0065-2504(03)34002-4.

Harvey, Jill E., Marko Smiljanić, Tobias Scharnweber, Allan Buras, Anna Cedro, Roberto Cruz-García, Igor Drobyshev, et al. 2020. ‘Tree Growth Influenced by Warming Winter Climate and Summer Moisture Availability in Northern Temperate Forests’. *Global Change Biology* 26 (4): 2505–18. https://doi.org/10.1111/gcb.14966.

Heidrich, Lea, Soyeon Bae, Shaun Levick, Sebastian Seibold, Wolfgang Weisser, Peter Krzystek, Paul Magdon, et al. 2020. ‘Heterogeneity–Diversity Relationships Differ between and within Trophic Levels in Temperate Forests’. *Nature Ecology & Evolution* 4 (9): 1204–12. https://doi.org/10.1038/s41559-020-1245-z.

Heine, Peggy, Jonas Hausen, Richard Ottermanns, Andreas Schäffer, and Martina Roß-Nickoll. 2019. ‘Forest Conversion from Norway Spruce to European Beech Increases Species Richness and Functional Structure of Aboveground Macrofungal Communities’. *Forest Ecology and Management* 432: 522–33. https://doi.org/10.1016/j.foreco.2018.09.012.

Herrmann, Steffen, Tiemo Kahl, and Jürgen Bauhus. 2015. ‘Decomposition Dynamics of Coarse Woody Debris of Three Important Central European Tree Species’. *Forest Ecosystems* 2 (1): 27. https://doi.org/10.1186/s40663-015-0052-5.

Hilmers, Torben, Nicolas Friess, Claus Bässler, Marco Heurich, Roland Brandl, Hans Pretzsch, Rupert Seidl, et al. 2018. ‘Biodiversity along Temperate Forest Succession’. *Journal of Applied Ecology* 55 (6): 2756–66. https://doi.org/10.1111/1365-2664.13238.

Hoppe, Björn, Witoon Purahong, Tesfaye Wubet, Tiemo Kahl, Jürgen Bauhus, Tobias Arnstadt, Martin Hofrichter, et al. 2016. ‘Linking Molecular Deadwood-Inhabiting Fungal Diversity and Community Dynamics to Ecosystem Functions and Processes in Central European Forests’. *Fungal Diversity* 77 (1): 367–79. https://doi.org/10.1007/s13225-015-0341-x.

Kahl, Tiemo, Tobias Arnstadt, Kristin Baber, Claus Bässler, Jürgen Bauhus, Werner Borken, François Buscot, et al. 2017. ‘Wood Decay Rates of 13 Temperate Tree Species in Relation to Wood Properties, Enzyme Activities and Organismic Diversities’. *Forest Ecology and Management* 391: 86–95. https://doi.org/10.1016/j.foreco.2017.02.012.

Karger, Dirk Nikolaus, Olaf Conrad, Jürgen Böhner, Tobias Kawohl, Holger Kreft, Rodrigo Wilber Soria-Auza, Niklaus E. Zimmermann, et al. 2017. ‘Climatologies at High Resolution for the Earth’s Land Surface Areas’. *Scientific Data* 4 (1): 170122. https://doi.org/10.1038/sdata.2017.122.

Larrieu, Laurent, Frédéric Gosselin, Frédéric Archaux, Richard Chevalier, Gilles Corriol, Emmanuelle Dauffy-Richard, Marc Deconchat, et al. 2019. ‘Assessing the Potential of Routine Stand Variables from Multi-Taxon Data as Habitat Surrogates in European Temperate Forests’. *Ecological Indicators* 104: 116–26. https://doi.org/10.1016/j.ecolind.2019.04.085.

Lasky, Jesse R., María Uriarte, Vanessa K. Boukili, David L. Erickson, W. John Kress, and Robin L. Chazdon. 2014. ‘The Relationship between Tree Biodiversity and Biomass Dynamics Changes with Tropical Forest Succession’. *Ecology Letters* 17 (9): 1158–67. https://doi.org/10.1111/ele.12322.

Lassauce, Aurore, Yoan Paillet, Hervé Jactel, and Christophe Bouget. 2011. ‘Deadwood as a Surrogate for Forest Biodiversity: Meta-Analysis of Correlations between Deadwood Volume and Species Richness of Saproxylic Organisms’. *Ecological Indicators* 11 (5): 1027–39. https://doi.org/10.1016/j.ecolind.2011.02.004.

Lee, Yong-Ju, Go-Eun Park, Hae-In Lee, and Chang-Bae Lee. 2024. ‘Stand Age-Driven Tree Size Variation and Stand Type Regulate Aboveground Biomass in Alpine-Subalpine Forests, South Korea’. *Science of The Total Environment* 915: 170063. https://doi.org/10.1016/j.scitotenv.2024.170063.

Lohr, Steven M., Sidney A. Gauthreaux, and John C. Kilgo. 2002. ‘Importance of Coarse Woody Debris to Avian Communities in Loblolly Pine Forests’. *Conservation Biology* 16 (3): 767–77. https://doi.org/10.1046/j.1523-1739.2002.01019.x.

Lutz, James A., Tucker J. Furniss, Daniel J. Johnson, Stuart J. Davies, David Allen, Alfonso Alonso, Kristina J. Anderson-Teixeira, et al. 2018. ‘Global Importance of Large-diameter Trees’. *Global Ecology and Biogeography* 27 (7): 849–64. https://doi.org/10.1111/geb.12747.

Lutz, James A., Andrew J. Larson, Mark E. Swanson, and James A. Freund. 2012. ‘Ecological Importance of Large-Diameter Trees in a Temperate Mixed-Conifer Forest’. *PLoS ONE* 7 (5): e36131. https://doi.org/10.1371/journal.pone.0036131.

Lynch, James F., and Dennis F. Whigham. 1984. ‘Effects of Forest Fragmentation on Breeding Bird Communities in Maryland, USA’. *Biological Conservation* 28 (4): 287–324. https://doi.org/10.1016/0006-3207(84)90039-9.

McGee, Gregory G., Donald J. Leopold, and Ralph D. Nyland. 1999. ‘Structural Charateristics of Old-Growth, Maturing, and Partially Cut Northern Hardwood Forests’. *Ecological Applications* 9 (4): 1316–29. https://doi.org/10.1890/1051-0761(1999)009%255B1316:SCOOGM%255D2.0.CO;2.

Mildrexler, David J., Logan T. Berner, Beverly E. Law, Richard A. Birdsey, and William R. Moomaw. 2020. ‘Large Trees Dominate Carbon Storage in Forests East of the Cascade Crest in the United States Pacific Northwest’. *Frontiers in Forests and Global Change* 3: 594274. https://doi.org/10.3389/ffgc.2020.594274.

Müller, Jörg, Herve Brustel, Antoine Brin, Heinz Bussler, Christophe Bouget, Elisabeth Obermaier, Ina M. M. Heidinger, et al. 2015. ‘Increasing Temperature May Compensate for Lower Amounts of Dead Wood in Driving Richness of Saproxylic Beetles’. *Ecography* 38 (5): 499–509. https://doi.org/10.1111/ecog.00908.

Oettel, Janine, Katharina Lapin, Georg Kindermann, Herfried Steiner, Karl-Manfred Schweinzer, Georg Frank, and Franz Essl. 2020. ‘Patterns and Drivers of Deadwood Volume and Composition in Different Forest

Types of the Austrian Natural Forest Reserves'. *Forest Ecology and Management* 463: 118016. https://doi.org/10.1016/j.foreco.2020.118016.
Økland, Bjørn, Alf Bakke, Sigmund Hågvar, and Torstein Kvamme. 1996. 'What Factors Influence the Diversity of Saproxylic Beetles? A Multiscaled Study from a Spruce Forest in Southern Norway'. *Biodiversity and Conservation* 5 (1): 75–100. https://doi.org/10.1007/BF00056293.
Otsu, Chiaki, Hayato Iijima, Takuo Nagaike, and Yoshinobu Hoshino. 2023. 'Responses to Changes in Precipitation of Plant Species Vary by Functional Groups on Understories of Temperate Forests in Central Japan'. *Forest Ecology and Management* 529: 120716. https://doi.org/10.1016/j.foreco.2022.120716.
Parisi, Francesco, Mirko Di Febbraro, FFabio Lombardi, Alessandro B. Biscaccianti, Alessandro Campanaro, Roberto Tognetti, and Marco Marchetti. 2019. 'Relationships between Stand Structural Attributes and Saproxylic Beetle Abundance in a Mediterranean Broadleaved Mixed Forest'. *Forest Ecology and Management* 432: 957–66. https://doi.org/10.1016/j.foreco.2018.10.040.
Pretzsch, Hans, and Torben Hilmers. 2024. 'Structural Diversity and Carbon Stock of Forest Stands: Tradeoff as Modified by Silvicultural Thinning'. *European Journal of Forest Research*, ahead of print, May 3. https://doi.org/10.1007/s10342-024-01691-z.
Pretzsch, Hans, and Gerhard Schütze. 2014. 'Size-Structure Dynamics of Mixed versus Pure Forest Stands'. *Forest Systems* 23 (3): 560. https://doi.org/10.5424/fs/2014233-06112.
Pretzsch, Hans, and Gerhard Schütze. 2016. 'Effect of Tree Species Mixing on the Size Structure, Density, and Yield of Forest Stands'. *European Journal of Forest Research* 135 (1): 1–22. https://doi.org/10.1007/s10342-015-0913-z.
Rappa, Nolan J., Michael Staab, Julian Frey, Nathalie Winiger, and Alexandra-Maria Klein. 2022. 'Multiple Forest Structural Elements Are Needed to Promote Beetle Biomass, Diversity and Abundance'. *Forest Ecosystems* 9: 100056. https://doi.org/10.1016/j.fecs.2022.100056.
Rieker, Daniel, Franz-S. Krah, Martin M. Gossner, Britta Uhl, Didem Ambarli, Kristin Baber, François Buscot, et al. 2022. 'Disentangling the Importance of Space and Host Tree for the Beta-Diversity of Beetles, Fungi, and Bacteria: Lessons from a Large Dead-Wood Experiment'. *Biological Conservation* 268: 109521. https://doi.org/10.1016/j.biocon.2022.109521.
Rohner, Brigitte, Harald Bugmann, and Christof Bigler. 2013. 'Estimating the Age–Diameter Relationship of Oak Species in Switzerland Using Nonlinear Mixed-Effects Models'. *European Journal of Forest Research* 132 (5–6): 751–64. https://doi.org/10.1007/s10342-013-0710-5.
Sabatini, Francesco Maria, Rafael Barreto de Andrade, Yoan Paillet, Péter Ódor, Christophe Bouget, Thomas Campagnaro, Frédéric Gosselin, et al. 2019. 'Trade-offs between Carbon Stocks and Biodiversity in European Temperate Forests'. *Global Change Biology* 25 (2): 536–48. https://doi.org/10.1111/gcb.14503.
Sandström, Jennie, Claes Bernes, Kaisa Junninen, Asko Lõhmus, Ellen Macdonald, Jörg Müller, and Bengt Gunnar Jonsson. 2019. 'Impacts of Dead Wood Manipulation on the Biodiversity of Temperate and Boreal Forests. A Systematic Review'. *Journal of Applied Ecology* 56 (7): 1770–81. https://doi.org/10.1111/1365-2664.13395.
Sturtevant, Brian R., John A. Bissonette, James N. Long, and Dave W. Roberts. 1997. 'Coarse Woody Debris as a Function of Age, Stand Structure, and Disturbance in Boreal Newfoundland'. *Ecological Applications* 7 (2): 702–12. https://doi.org/10.1890/1051-0761(1997)007%255B0702:CWDAAF%255D2.0.CO;2.
Sullivan, Martin J. P., Joey Talbot, Simon L. Lewis, Oliver L. Phillips, Lan Qie, Serge K. Begne, Jerôme Chave, et al. 2017. 'Diversity and Carbon Storage across the Tropical Forest Biome'. *Scientific Reports* 7 (1): 39102. https://doi.org/10.1038/srep39102.
Thorn, Simon, Bernhard Förster, Christoph Heibl, Jörg Müller, and Claus Bässler. 2018. 'Influence of Macroclimate and Local Conservation Measures on Taxonomic, Functional, and Phylogenetic Diversities of Saproxylic Beetles and Wood-Inhabiting Fungi'. *Biodiversity and Conservation* 27 (12): 3119–35. https://doi.org/10.1007/s10531-018-1592-0.
Tinya, Flóra, Bence Kovács, András Bidló, Bálint Dima, Ildikó Király, Gergely Kutszegi, Ferenc Lakatos, et al. 2021. 'Environmental Drivers of Forest Biodiversity in Temperate Mixed Forests – A Multi-Taxon Approach'. *Science of The Total Environment* 795: 148720. https://doi.org/10.1016/j.scitotenv.2021.148720.
Wu, Donghao, Sebastian Seibold, Katherina A. Pietsch, M.D. Farnon Ellwood, and Mingjian Yu. 2023. 'Tree Species Richness Increases Spatial Variation but Not Overall Wood Decomposition'. *Soil Biology and Biochemistry* 183: 109060. https://doi.org/10.1016/j.soilbio.2023.109060.
Zeller, Laura, Agnes Förster, Constanze Keye, Peter Meyer, Christian Roschak, and Christian Ammer. 2023. 'What Does Literature Tell Us about the Relationship between Forest Structural Attributes and Species

Richness in Temperate Forests? – A Review'. *Ecological Indicators* 153: 110383. https://doi.org/10.1016/j.ecolind.2023.110383.

Zellweger, Florian, Andri Baltensweiler, Christian Ginzler, Tobias Roth, Veronika Braunisch, Harald Bugmann, and Kurt Bollmann. 2016. 'Environmental Predictors of Species Richness in Forest Landscapes: Abiotic Factors versus Vegetation Structure'. *Journal of Biogeography* 43 (6): 1080–90. https://doi.org/10.1111/jbi.12696.

Zhang, Jian, W. Daniel Kissling, and Fangliang He. 2013. 'Local Forest Structure, Climate and Human Disturbance Determine Regional Distribution of Boreal Bird Species Richness in Alberta, Canada'. *Journal of Biogeography* 40 (6): 1131–42. https://doi.org/10.1111/jbi.12063.
